# Do all psychological treatments really work the same in posttraumatic stress disorder?

**DOI:** 10.1016/j.cpr.2009.12.001

**Published:** 2010-03

**Authors:** Anke Ehlers, Jonathan Bisson, David M. Clark, Mark Creamer, Steven Pilling, David Richards, Paula P. Schnurr, Stuart Turner, William Yule

**Affiliations:** a King's College London, Institute of Psychiatry, UK; b Cardiff University School of Medicine, Department of Psychological Medicine, UK; c NIHR Biomedical Research Centre for Mental Health, South London & Maudsley NHS Trust and King's College London, UK; d Australian Centre for Posttraumatic Mental Health, Department of Psychiatry, Australia; e University College London, and Centre for Outcomes, Research & Effectiveness, UK; f University of Exeter, School of Psychology, UK; g Veterans Affairs National Center for PTSD and Dartmouth Medical School, USA; h Trauma Clinic, London, UK

**Keywords:** Posttraumatic stress disorder, Meta-analysis, Cognitive behavior therapy, EMDR, Psychotherapy, Clinical trials

## Abstract

A recent meta-analysis by Benish, Imel, and Wampold (2008, *Clinical Psychology Review*, *28*, 746–758) concluded that all *bona fide* treatments are equally effective in posttraumatic stress disorder (PTSD). In contrast, seven other meta-analyses or systematic reviews concluded that there is good evidence that trauma-focused psychological treatments (trauma-focused cognitive behavior therapy and eye movement desensitization and reprocessing) are effective in PTSD; but that treatments that do not focus on the patients' trauma memories or their meanings are either less effective or not yet sufficiently studied. International treatment guidelines therefore recommend trauma-focused psychological treatments as first-line treatments for PTSD. We examine possible reasons for the discrepant conclusions and argue that (1) the selection procedure of the available evidence used in Benish et al.'s (2008)meta-analysis introduces bias, and (2) the analysis and conclusions fail to take into account the need to demonstrate that treatments for PTSD are more effective than natural recovery. Furthermore, significant increases in effect sizes of trauma-focused cognitive behavior therapies over the past two decades contradict the conclusion that content of treatment does not matter. To advance understanding of the optimal treatment for PTSD, we recommend further research into the active mechanisms of therapeutic change, including treatment elements commonly considered to be non-specific. We also recommend transparency in reporting exclusions in meta-analyses and suggest that *bona fide* treatments should be defined on empirical and theoretical grounds rather than by judgments of the investigators' intent.

## 1 Introduction

Meta-analyses of treatments for posttraumatic stress disorder (PTSD) have concluded that trauma-focused psychological treatments, such as individual trauma-focused cognitive behavior therapy (TFCBT) and eye movement desensitization and reprocessing (EMDR), are efficacious (Australian Centre for Posttraumatic Mental Health, 2007; Bisson & Andrew, 2009; Bisson et al., 2007; Bradley, Greene, Russ, Dutra, & Westen, 2005; Cloitre; 2009; Seidler & Wagner, 2006; van Etten & Taylor, 1998). These treatments have in common a focus on the patients' memories of their traumatic events and the personal meanings of the trauma. Meta-analyses have consistently found that there is no difference in efficacy between different forms of these trauma-focused treatments (Australian Centre for Posttraumatic Mental Health, 2007; Bisson & Andrew, 2009; Bisson et al., 2007; Bradley et al., 2005; Seidler & Wagner, 2006). Current treatment guidelines therefore recommend several trauma-focused psychological treatments as first-line treatments for PTSD (American Psychiatric Association, 2004; Australian Centre for Posttraumatic Mental Health, 2007; Foa, Keane, Friedman, & Cohen, 2005; National Institute of Clinical Excellence, 2005; Stein et al., 2009; Veterans Health Administration & Department of Defense, 2004).^1^

A range of other PTSD treatments has also been studied, albeit less frequently. These include various stress-management programs (e.g., Carlson, Chemtob, Resnka, Hedlund, & Muraoka, 1998; Foa, Rothbaum, Riggs, & Murdock, 1991; Vaughan et al., 1994), supportive (Rogerian, non-directive) therapy (e.g., Blanchard et al., 2003), hypnotherapy (Brom, Kleber, & Defares, 1989), psychodynamic (Brom et al., 1989), and interpersonal therapy (Krupnick et al., 2008). Meta-analyses and systematic reviews have considered the magnitude of symptom change with treatment and/or head-to-head comparisons of different treatments and have concluded that non-trauma-focused treatments tend to be less efficacious in treating PTSD than trauma-focused treatments, or have not been studied sufficiently to determine their effectiveness (Australian Centre for Posttraumatic Mental Health, 2007; Bisson & Andrew, 2009; Bisson et al., 2007; Bradley et al., 2005; Committee on Treatment of Posttraumatic Stress Disorder, Institute of Medicine of the National Academies, 2008^2^; Stein et al., 2009; van Etten & Taylor, 1998; see also Ramchandani & Jones, 2003, for similar conclusions for sexually abused children).

A recent meta-analysis by Benish et al. (2008) comes to a dramatically different conclusion. Unlike earlier meta-analyses that categorized treatments by treatment methods, treatments were categorized on the basis of whether they were judged to be “intended to be therapeutic,” or *bona fide* treatments. Benish et al. (2008) selected a subset of the head-to-head comparisons between different psychological treatments included in previous meta-analyses deemed “intended to be therapeutic,” using criteria suggested by Wampold et al. (1997). The distribution of differences in outcome between different treatments across the selected studies was then analyzed. As the effect sizes for differences between treatments were homogenously distributed around zero, Benish et al. (2008) concluded that all *bona fide* treatments are equally effective in PTSD.

This paper examines reasons for the discrepant conclusions of the meta-analyses: Does it matter whether treatments are trauma-focused or not, as current treatment guidelines suggest (Australian Centre for Posttraumatic Mental Health, 2007; Committee on Treatment of Posttraumatic Stress Disorder, Institute of Medicine of the National Academies, 2008; National Institute of Clinical Excellence, 2005; Stein et al., 2009) or is any therapy that is intended to be therapeutic equally effective in PTSD, as Benish et al. (2008) suggest? We critically re-examine the evidence presented by Benish et al. (2008) and suggest ways to test which of the interpretations of the currently available evidence is correct.

We argue that (1) the selection procedure of the available evidence used in Benish et al.'s (2008) meta-analysis introduces bias; and (2) the analysis and conclusions fail to take into account the need to demonstrate that treatments for PTSD are more effective than natural recovery. Furthermore, significant increases in effect sizes of trauma-focused cognitive behavior therapies over the past two decades contradict the conclusion that content of treatment does not matter.

We then make suggestions to help advance understanding of the optimal treatment for PTSD. These include (a) further research into the active mechanisms of therapeutic change, including treatment elements commonly considered to be non-specific, (b) transparency in reporting exclusions in meta-analyses, and (c) defining *bona fide* treatments on empirical and theoretical grounds rather than by judgments of the investigators' intent.

## 2 Selection introduces bias. The example of non-directive therapies

The Benish et al. (2008) meta-analysis excluded a large number of the comparisons from randomized controlled trials included in the previous meta-analyses. Only 17 comparisons from 15 studies remained. In comparison, Cloitre's (2009) review lists 44 head-to-head comparisons of face-to-face treatment from 27 studies that were published up to early 2007, the time period reviewed by Benish et al. (2008). Benish et al. state that their search of the literature identified 26 comparisons from 22 studies. This raises the question of whether selection procedures in the Benish et al. (2008) study may have introduced bias. We will examine this question by looking at the way the meta-analysis dealt with non-directive therapies.

Supportive (non-directive, Rogerian, person-centered) therapy is currently widely offered to patients with PTSD in clinical practice. In the British National Health Service, it is the treatment most commonly offered to PTSD patients identified in primary care (e.g., Ehlers, Gene-Cos, & Perrin, 2009). It is also widely practiced in the United States. Pingitore, Scheffler, Haley, Sentell, and Schwalm (2001) found that 58% of psychologists practicing in California reported that they provided supportive psychotherapy. There is a good rationale for using supportive therapy to treat PTSD as social support has been shown to be one of the best predictors of recovery in PTSD (Ozer, Best, Lipzey, & Weiss, 2003). It is, therefore, surprising that most of the trials using such therapies were excluded from the Benish et al. (2008) meta-analysis. The authors justified the exclusion by arguing that the treatments used in the trials were “not intended to be therapeutic.” This judgment was made even if the trial showed that the treatment was effective (i.e., superior to a no treatment control condition, e.g., Blanchard et al., 2003).

**Fig. 1:**
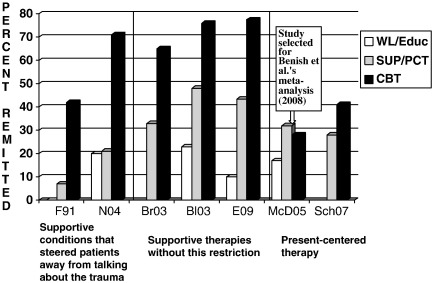
Comparison of individual non-directive treatments with trauma-focused CBT programs. Intent-to-treat analyses for percent remitted (loss of PTSD diagnosis) with treatment. The study marked with an arrow was selected for Benish et al.'s (2008) meta-analysis. Abbreviations: WL/EDU = waitlist or psychoeducation; SUP/PCT = supportive or present-centred therapy; CBT = trauma-focused CBT. Bl03 = Blanchard et al. (2003). Br03 = Bryant et al. (2003). E09 = Ehlers et al. (2009). F91 = Foa et al. (1991). McD05 = McDonagh et al. (2005). N04 = Neuner et al. (2004). Sch07 = Schnurr et al. (2007).

In trials of non-directive treatments, two different labels were used to describe the treatment conditions, (1) supportive therapy or supportive counseling and (2) present-centered therapy. All trials using the former label were excluded from the Benish et al. (2008) meta-analysis. The authors use Foa et al.'s (1991) study to justify the argument that the supportive therapies used in the research trials were not intended to be therapeutic. In the Foa et al. study (and in a study by Neuner, Schauer, Klaschik, Karunakara, & Elbert, 2004), therapists in the supportive counseling condition were instructed to steer patients away from talking about their specific traumatic events. We agree that this would not necessarily be representative of supportive therapy as it would be delivered by a practicing clinician, and may therefore underestimate the effect of counseling. However, this restriction did not apply to two other excluded studies (Blanchard et al., 2003; Bryant, Moulds, Guthrie, Dang, & Nixon, 2003). As shown in Fig. 1, these two studies (but not the Foa et al., 1991, and Neuner et al., 2004, studies) showed substantial recovery rates with supportive therapy, but nevertheless found that supportive therapy was less effective than TFCBT.

On the other hand, Benish et al. (2008) judged present-centered therapy (PCT) to be a *bona fide* treatment. This treatment aims to control for non-specific therapeutic factors common to active psychotherapies. Therapists help patients identify current life problems and discuss them in a supportive, non-directive mode. PCT as delivered in the trials included an explicit rationale for focusing on the present, psychoeducation about PTSD symptoms, and homework assignments (e.g., Schnurr et al., 2007). Many of these treatment components were also included in the supportive therapies excluded from the Benish et al. meta-analysis (e.g., Blanchard et al., 2003). The reasons for the classification difference remain unclear.

Benish et al.'s (2008) meta-analysis included two studies comparing PCT and TFCBT, a study of individual treatment of survivors of childhood sexual abuse (CSA, McDonagh et al., 2005; see Fig. 1), and a study of *group* therapy for Vietnam veterans (Schnurr et al., 2003). Both of these patient populations differ in a number of respects from those included in most other randomized controlled trials of PTSD treatments in that the patients had experienced multiple and prolonged traumas that happened many years ago, are often considered difficult-to-treat, and may require additional interventions (Cloitre, 2009). In the Schnurr et al. (2003) Vietnam veterans study, both group treatments led to modest symptom change in the overall intent-to-treat analysis, which included participants who did not receive any treatment. Schnurr et al. (2003) also presented an analysis of patients who received an adequate dose of group therapy (at least 24 sessions). This analysis suggested that “TFGT (*trauma-focused group therapy*) was better than PCGT (*present*-*centered group therapy*) for treating avoidance and numbing, and possibly, overall PTSD symptoms.” (p. 487). Either way, the fact that this was a group-based treatment makes it difficult to compare with the other studies that were restricted to individual treatment.

In the childhood sexual assault sample (McDonagh et al., 2005), both individual TFCBT and PCT were superior to the wait list condition at post treatment. In addition, the results pointed to an advantage of CBT at the 3-months follow-up in the completer analysis in that TFCBT participants (82%) were significantly more likely than those receiving PCT (42%) to no longer meet criteria for a PTSD diagnosis.

The pattern of results points to several possible interpretations. One possibility is, as Benish et al. (2008) suggest, that PCT is as effective as TFCBT. The second possibility is that the lack of differences in the intent-to-treat analyses in the McDonagh et al. (2005) and Schnurr et al. (2003) studies may be a function of the difficult-to-treat multiple trauma populations studied.

If Benish et al. (2008) are correct and PCT is equivalent to TFCBT, then one would predict further comparisons of these treatments in other patient populations to show equivalence. If current PTSD treatment guidelines are correct and trauma-focus matters, then one would predict further comparisons to show that TFCBT is superior.

Two recent trials are relevant for deciding between these hypotheses. First, a further trial comparing PCT and TFCBT was omitted from Benish et al.'s (2008) meta-analysis. Schnurr et al. (2007) compared PCT with Prolonged Exposure (a form of TFCBT) in female veterans with PTSD. This study found that TFCBT was more effective than PCT (see Fig. 1). Another trial (Ehlers et al., in preparation) that was not available at the time of the review compared emotion-focused supportive therapy, which shares many of the active elements of PCT and allowed patients to decide what they wanted to talk about, with Cognitive Therapy for PTSD (a form of TFCBT). Emotion-focused supportive therapy was shown to the effective (superior to wait list). Nevertheless, TFCBT was superior (see Fig. 1). Thus, both of these studies are at odds with Benish et al.'s (2008) conclusions and in line with the interpretation that trauma-focus matters and therefore further support the conclusions of recent treatment guidelines that trauma-focused psychological treatments have an advantage over non-directive treatments (Australian Centre for Posttraumatic Mental Health, 2007; National Institute of Clinical Excellence, 2005; Stein et al., 2009).

In summary, when one considers all studies comparing individual non-directive therapies with individual TFCBT, it is clear that TFCBT performs better (see Fig. 1). By excluding nearly all of these studies, Benish et al. (2008) arrive at the conclusion that there is no difference. Further selectivity is evident in the quotes from published studies reproduced in the paper to support of the equal efficacy argument. Table 1 illustrates this point.

## 3 Need to show that recommended treatments are more effective than no treatment: the case of hypnotherapy and psychodynamic therapy

Previous meta-analyses have found that there are no differences in efficacy between different versions of trauma-focused psychological treatments (Australian Centre for Posttraumatic Mental Health, 2007; Bisson et al., 2007; Bradley et al., 2005; Cloitre; 2009; Seidler & Wagner, 2006; van Etten & Taylor, 1998). Of the 15 trials included in Benish et al.'s (2008) meta-analysis, 9 are comparisons between different versions of trauma-focused treatments (e.g., exposure vs. cognitive therapy and exposure vs. EMDR; Devilly & Spence, 1999; Ironson, Freund, Strauss, & Williams, 2002; Marks, Lovell, Noshirvani, Livanou, & Thrasher, 1998; Paunovic & Öst, 2001; Power et al., 2002; Resick, Nishith, Weaver, Astin, & Feuer, 2002; Rothbaum, Astin, & Marsteller, 2005; Tarrier et al., 1999; Taylor et al., 2003). Thus, one interpretation of Benish et al.'s (2008) study is that it replicated the result of previous meta-analyses that different forms of trauma-focused psychological treatments have similar effects.

Benish et al. (2008), however, wish to extend the equal efficacy conclusion to all treatments judged to be *bona fide.* The data set in the Benish et al. (2008) analysis includes only 6 trials that studied treatments other than TFCBT or EMDR, and some treatments were only represented by one study (Brom et al., 1989: trauma desensitization vs. psychodynamic therapy vs. hypnotherapy: Foa et al., 1991, 1999: Prolonged Exposure vs. stress inoculation; Lee, Gavriel, Drummond, Richards, and Greenwald, 2002: EMDR vs. stress inoculation; McDonagh et al., 2005: individual CBT vs. present-centered therapy; Schnurr et al., 2003: Trauma-focused group therapy vs. present-centered group therapy). This is a very small database for reaching conclusions about these interventions.

One important consideration in evaluating the findings of these 6 studies is that the result that two interventions did not differ in a particular study does not necessarily mean that the treatments are effective. This is because comparisons between different non-effective treatments will also produce a null difference finding. If a meta-analysis of different drugs used to treat bacterial infections mainly included no difference comparisons between antibiotics, but also a few no difference studies comparing aspirin with vitamin C, one would not conclude that antibiotics, aspirin, and vitamin C are all similarly effective in treating infections. Concluding equivalence of individual treatments from no difference in a mixture of relevant and irrelevant comparisons is misleading.

Furthermore, in medicine, there has been increasing awareness that null findings in small studies cannot be interpreted as demonstrating equivalence between treatments, and that establishing noninferiority or equivalence of a new treatment requires trials specifically designed for that purpose (Blackwelder, 1982; Greene, Concato & Feinstein, 2000; Jones, Jarvis, Lewis, & Elbutt, 1996; Le Henanff, Giraudeau, Baron, & Ravaud, 2006; Piaggio, Elbourne, Altman, Pocock, & Evans, 2006). The new treatment needs to be directly compared with an established treatment in an adequately powered trial (e.g., Piaggio et al., 2006). Similar considerations apply to psychotherapy research (Greene, Morland, Durkalski, & Frueh, 2008).

Thus, lack of difference between two treatments in a given study needs to be interpreted in the context of overall effect sizes and comparisons against no treatment. Benish et al.'s meta-analysis (2008) fails to take this into account. The need to demonstrate that an intervention is more effective than no intervention (Stevens, Hynan, & Allen, 2000) is especially relevant in PTSD as it is well established that this disorder shows substantial natural recovery (Kessler, Sonnega, Bromet, Hughes, & Nelson, 1995). Treatments should only be recommended if they lead to greater improvement than what can be expected from natural recovery. Even if patients improve with treatment, this can represent a harmful rather than beneficial effect of the intervention, depending on the rate of natural recovery in the population (Ehlers & Clark, 2003). One example where this problem became evident is psychological debriefing, which was widely used as an early intervention provided to everyone involved in a trauma, regardless of symptoms, as it was believed to prevent PTSD. Debriefing has a plausible rationale and has been used with the intention to be therapeutic (thus a *bona fide* intervention). However, when randomized controlled trials of single sessions of individual debriefing were conducted, it became clear that — contrary to the investigators' hypotheses — the intervention did not lead to greater reductions in PTSD symptoms compared to no treatment, and alarmingly, in some studies even made patients worse (Bisson, Jenkins, Alexander, & Bannister, 1997; Mayou, Ehlers, & Hobbs, 2000; Rose & Bisson, 1998). Current guidelines therefore advise against single session posttrauma interventions that ask survivors to give detailed accounts of their traumatic experience (Australian Centre for Posttraumatic Mental Health, 2007; Committee on Treatment of Posttraumatic Stress Disorder, Institute of Medicine of the National Academies, 2008; National Institute of Clinical Excellence, 2005; Stein et al., 2009).

The argument that treatments need to be shown to be more effective than no intervention is relevant for Wampold's (2008) conclusion that the Benish et al. (2008) analysis showed that hypnotherapy and psychodynamic therapy are effective treatments for PTSD. The evidence comes from one study by Brom et al. (1989). These authors compared hypnotherapy and psychodynamic therapy with trauma desensitization, an early form of exposure therapy. Participants had experienced a trauma in adulthood, most commonly the loss of a loved one through suicide or murder. In this study, neither hypnotherapy nor psychodynamic therapy was consistently more effective than the wait list control condition across the analyses used (Brom et al., 1989, p. 610). In addition, Brom et al. (1989) pointed out that patients in psychodynamic therapy showed slower overall change than those in the other two treatment conditions, and did *not* improve in intrusive symptoms significantly, regardless of analysis method. Interestingly, Brom et al. (1989) attributed the effect of hypnotherapy and trauma desensitization on intrusions to the fact that both treatments addressed trauma memories, whereas the psychodynamic treatment did not — and thus anticipated the conclusions of recent meta-analyses that suggest a focus on trauma memories is important. Table 1 contrasts the statements extracted by Benish et al. (2008) with the full quotes from this paper.

**Fig. 2:**
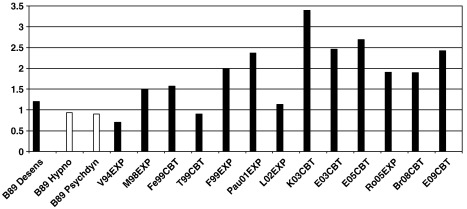
Effect sizes for changes in PTSD symptoms with treatment for Brom et al.'s (1989) study and for trauma-focused CBT programs (PTSD following trauma in adulthood). In line with Brom et al., effect sizes are based on completers. To ensure the comparison is fair, only studies with similar or lower drop-out rates as in Brom et al. are shown (drop-out rates in parentheses below). However, the pattern would be the same if all trials were shown. Effect sizes were calculated as the pre-post difference in PTSD symptom scores, divided by the pooled standard deviation. Abbreviations: EXP = exposure therapies, CBT = cognitive behavior therapies, Desens = Trauma desensitization, Hypno = hypnotherapy, Psychodyn = psychodynamic therapy; B89 = Brom et al. (1989, 11%). Br08 = Bryant et al. (2008, 17%). E03 = Ehlers et al. (2003, 0%), E05 = Ehlers, Clark, Hackmann, McManus, and Fennell (2005, 0%), E09 = Ehlers et al. (2009, 3%). Fe99 = Fecteau and Nicki (1999, 17%). F99 = Foa et al. (1999, 8%). K03 = Kubany, Hill and Owens (2003; 5%). M98 = Marks et al. (1998, 13%). L02 = Lee et al. (2002, 8%). Ro05 = Rothbaum et al. (2005, 13%). T99 = Tarrier et al. (1999, 11%). V94 = Vaughan et al. (1994, 8%).

It is also noteworthy that the early exposure program used in the Brom et al. (1989) study is no longer widely used. Current TFCBT programs achieve greater improvement with treatment. In a recent meta-analysis of PTSD trials of TFCBT, Öst (2008) found a significant positive correlation of *r* = .34 between effect size and year of publication for PTSD, indicating a significant improvement with time (see also Fig. 2). This finding contradicts Benish et al.'s (2008) conclusion that type of treatment does not matter. It also makes averaging the results of studies over time problematic.

Fig. 2 shows the pre-post treatment effect sizes for psychodynamic treatment and hypnotherapy observed in Brom al.'s (1989) study and those of TFCBT programs in older and recent randomized controlled trials. Brom et al. (1989) only report completer data for a self-report measure of PTSD symptoms. Completer data have the problem that drop-out rates vary across studies and may not be random. For comparability, Fig. 2 therefore only shows effect sizes from studies with similar drop-out rates. However, the pattern would be the same if all TFCBT trials were shown. The effect sizes in Fig. 2 do not seem to fit well with Benish et al.'s (2008) conclusion that hypnotherapy and psychodynamic treatment are as effective as recent TFCBT programs; however, direct comparisons are needed before conclusions are drawn.

Contrary to Wampold's (2008) conclusion, it appears premature to conclude from a single study with mixed results that psychodynamic therapy and hypnotherapy are effective in PTSD. Further evidence is needed before these treatments are widely used for treating PTSD. The finding that psychodynamic therapy did not improve intrusive reexperiencing, the core symptom of PTSD, suggests caution in recommending its use until efficacy is demonstrated in further trials.

## 4 A way forward

### 4.1 Need for properly powered superiority and equivalence trials

Meta-analyses oversimplify matters. They require arbitrary decisions about categories and inclusions and exclusions, and these decisions influence the results. We have outlined the reasons why Benish et al.'s (2008) meta-analysis is selective and why we believe that the authors, and Wampold (2008), incorrectly interpret the available evidence when they conclude that all *bona fide* treatments for PTSD are equally effective. We have also shown that a more detailed look at the evidence cited by the authors remains consistent with the conclusion of previous meta-analyses and current treatment guidelines that psychological treatments that focus on the trauma have advantages over treatments that do not. Furthermore, we have shown that even the studies cited in support of Benish et al.'s (2008) no difference conclusion present some evidence for an advantage of trauma focus.

We concede that previous meta-analyses (e.g., Bisson et al., 2007; Bradley et al., 2005) also necessitated arbitrary decisions about treatment categories. The heterogeneous category “other” used in previous meta-analyses (Bisson et al., 2007) reflects the fact that there is a lack of trials investigating the efficacy of treatments other than CBT or EMDR. “There have been ….very few or no good studies of a range of other psychotherapy interventions” (Stein et al., 2009). As this evidence grows, it will become possible to distinguish further between treatments that are effective and those that are not. The current evidence is too sparse for meaningful comparisons, other than to demonstrate the superiority of trauma-focused psychological treatments above those that do not focus on the trauma. Properly powered superiority and equivalence trials are needed to determine whether specific active treatments other than TFCBT and EMDR are effective in PTSD.

### 4.2 Transparency in reporting meta-analyses

The above discussion makes it clear that there is a need for greater transparency in reporting meta-analyses (Senn, 2009). We suggest that journals require authors of meta-analyses to include a flowchart of the studies identified and excluded (QUORUM statement, Moher et al., 1999), and report the effect sizes for the excluded studies. This would allow the reader to evaluate what influence the exclusion criteria had on effect sizes and conclusions. For example, in Benish et al.'s (2008) report, the reader cannot determine whether a RCT was excluded or simply missed in the search.

### 4.3 Standards for adequate treatment delivery in trials

The conclusiveness of meta-analyses depends on the quality of the trials that are included in the analysis. Some trials may be compromised by not delivering treatments to a sufficiently high standard for meaningful comparisons. The inclusion of such flawed trials in meta-analyses may lead to distorted or misleading results. Benish et al.'s (2008) meta-analysis attempts to address this problem by excluding trials that do not meet the Wampold et al. (1997) criteria for *bona fide* treatments.

However, we do not think that these criteria solve the problem of detecting flawed trials. Wampold et al.'s (1997) definition of *bona fide* is based on judgments of what the investigator thought when designing the trial. If an investigator used a treatment with the intention to control for common treatment factors, then it is assumed that the therapists working in the trial delivered this treatment in a way that is not therapeutic. We have discussed above that the evidence from PTSD trials does not support this view. Supportive therapies showed therapeutic effects despite Benish et al.'s (2008) judgment that they were not intended to be therapeutic and had to be excluded. Similarly, Smits and Hofmann (2009) found in their meta-analysis that the pre-post treatment effect size for non-specific treatments in PTSD was moderate (Hedges *g* = .50) and the average response rate was 34%, contradicting the assumption that these are not therapeutic.

There is also a conceptual problem with classifying treatments as “not intended to be therapeutic” on the grounds that they were used to control for non-specific elements of therapy. Common (non-specific) elements of psychotherapies are taught as basic skills in psychotherapy programs, presumably because these are universally seen as therapeutic. Examples include active listening, problem solving, encouraging self-reflection and coping, and providing emotional support. It remains unclear why and how these treatment components would become non-therapeutic when they are used in a trial. (We also find it hard to picture therapists that are trying not to be therapeutic when interacting with patients).

Thus, Wampold et al.'s (1997) criteria neither work well empirically nor conceptually in identifying poorly implemented treatments. There is increasing awareness in psychotherapy research that researchers need to demonstrate that the treatments were delivered competently. It is recommended that trials routinely report measures of treatment adherence, therapist competence and treatment credibility, as well as level of therapist training (e.g., Perepletchikova & Kazdin, 2005). This will allow the identification of poor quality trials in a less arbitrary way than the Wampold et al. (1997) criteria.

### 4.4 Criteria for active treatments

Another issue in the evaluation of the validity of clinical trials concerns the question of what treatments can reasonably be expected to be active treatments for a given disorder. The Benish et al. (2008) meta-analysis excluded groups of treatments because they were judged to be non-active on the basis of Wampold et al.'s (1997) criteria. Again, we think that these criteria give misleading answers.

Several therapies excluded by Benish et al. (2008) have a theoretical and empirical basis that would suggest they may be effective in PTSD. As shown above, supportive therapies excluded from Benish et al.'s (2008) meta-analysis included the non-specific elements of good therapy and they were not inert. Similarly, Benish et al. (2008) excluded all treatments that are variants of relaxation training, including biofeedback-assisted relaxation (e.g., Carlson et al., 1998; Taylor et al., 2003; Vaughan et al., 1994) although there is a good rationale for the use of relaxation training in PTSD as it targets hyperarousal, one of the core symptoms of the disorder, and although use of relaxation for treating anxiety and stress has a long tradition in cognitive behavior therapy and has been shown to be therapeutic across a range of anxiety disorders (Norton & Price, 2007; Öst, 2008).

As Schnurr (2007) described, psychotherapy trials are conducted with different purposes, and different designs allow different conclusions. Some trials are conducted to show that treatment effects are due to certain procedures rather than non-specific factors only. Benish et al.'s (2008) meta-analysis excluded most of these trials. The authors assumed that the Wampold et al. (1997) criteria objectively classify control treatments into ‘non-therapeutic’ (non-*bona fide*) controls and active controls (*bona fide*). This relies on the assumption that treatments that are judged to be not *bona fide* are less effective than those judged to be *bona fide* and thus dilute the effects of the comparison treatments. Three independent meta-analyses (Bowers & Clum, 1988; Hofmann & Smits, 2008; Stevens et al., 2000) have not supported this premise. Similarly, for PTSD, Cloitre's (2009) meta-analysis found that the non-directive therapies excluded from Benish et al.'s (2008) meta-analysis of PTSD treatments had larger modal effect sizes than the only form of these treatments that was included, present-centered therapy. Thus, the Wampold et al. (1997) criteria do not address the problem of identifying candidate active treatments in a convincing way. Benish et al.'s (2008) meta-analysis seems to be confusing “non-therapeutic” with “non-specific”.

We suggest that instead the definition of *bona fide* treatments should be based on empirical and theoretical considerations. If a treatment is shown to be more effective than no treatment, then it can be considered *empirically bona fide* for this disorder. If a treatment has been shown to be effective in other disorders, and there is a theoretically plausible rationale for why it may also work in the disorder under consideration, then it can be considered *theoretically bona fide* (but research may show that it does not work for this condition).

### 4.5 Treatment mechanisms

A promising way forward in identifying the best treatments for PTSD appears to be furthering the understanding of what mechanisms are involved in PTSD and need to be targeted in treatment. For TFCBT, work along these lines has lead to a refinement of treatment programs, which has led to improved effect sizes (Öst, 2008). It is likely that further refinements are possible. Similarly, it would be important to understand the mechanisms by which present-focused therapy and other forms of non-directive therapy work. The same is true for other therapies that do not focus on the trauma, such as interpersonal therapy (e.g., Krupnick et al., 2008) or stress-inoculation training (e.g., Foa et al., 1999). The present results suggest that a proportion of PTSD patients recover with these treatments, and thus do not appear to require systematic confrontation with their trauma memories. This is an interesting finding and, if the mechanisms were better understood, refinement of procedures that target these mechanisms in treatment may lead to improved outcomes.

In this context, it is important to note that the concept of placebo controls in psychological treatment trials is problematic (Schnurr, 2007). Psychological control treatments aim to control for non-specific elements of active treatments such as establishing a trusting relationship, emotional support, education about PTSD, mobilization of hope, giving a rationale, or homework assignments. When assessing effects of medication, the placebo effect is considered to reflect psychological mechanisms that are not relevant to the action of the drug. In psychological treatments, understanding the significance of the so-called non-specific effects is more complex. Some of the non-specific elements of psychological treatments may actually represent active mechanisms of change. For example, many patients with PTSD following interpersonal violence believe that they cannot trust anybody. Establishing a trusting relationship with the therapist can help shift this belief. Furthermore, some of the non-specific elements included in psychological control therapies, such as homework assignments, are not common to all psychological treatments as used in clinical practice. If they make a difference, and if it the mechanisms are understood, then such non-specific procedures could be used to enhance the effects of non-trauma-focused therapies.

## 5 Conclusion

Benish et al. (2008) conclude that treatments that are similar to the treatments included in their meta-analysis are equally efficacious — namely, treatments similar to “stress management, psychodynamic treatments, EMDR, hypnotherapy, cognitive behavioral treatment, exposure-based treatment, and treatments designed to explicitly exclude exposure (e.g., present-centered therapy)” (p. 755). We disagree. The experience with single sessions of psychological debriefing has shown that some well-intended treatments have null or even negative effects in trauma survivors and caution is indicated before applying unproven treatments widely. The conclusion that all treatments for PTSD are equally efficacious represents on overgeneralization from a biased selection of the available evidence (Benish et al., 2008; Wampold, 2008) and may have the unfortunate consequence that people suffering this serious and disabling condition will not be provided with the best available intervention. Treatments for PTSD should only be recommended for general use if they have been shown to be effective, not because averaging effects over many studies washes out differences.

Nevertheless, the available results also suggest that therapeutic elements common to many psychotherapies may be therapeutic, but less so than trauma-focused therapies. Understanding the mechanism of these non-specific factors may help improve available treatments. While the present state of the PTSD literature suggests that directly addressing trauma memories in the treatment of PTSD has an advantage over non-specific factors, further research may identify active ingredients among the non-specific factors.

We have shown above that Wampold et al.'s (1997) and Benish et al.'s (2008) approach to defining *bona fide* PTSD treatments by judging the investigators' intent does not relate well to the treatment outcomes observed in the trials and has conceptual problems. Some treatments worked despite Benish et al.'s (2008) judgment that they were *not intended to be therapeutic* (e.g., supportive therapies), and others were delivered with therapeutic intent, but did not work (e.g., single sessions of individual debriefing). We suggest that instead the definition of *bona fide* treatments should be based on empirical (*empirically bona fide*) and theoretical considerations (*theoretically bona fide).*

## Figures and Tables

**Table 1:** Comparison of results cited in Benish et al. (2008) with authors' conclusions for studies comparing trauma-focused cognitive behavior therapy with other treatments.

Quotes in Benish et al. (2008)	Authors' summary of results and conclusions
*Brom et al (1989)*	
“the differences between therapies are small” (p. 610) “Similarity of … treatment(s) … based on quite diverging theoretical considerations” (p. 610)	*The full quotes read*: “The treatments do benefit some in comparison with a control group…, but they do not benefit everyone, the effects are not always substantial, and the differences between therapies are small” (p. 610) “The similarity of the results in the three treatment conditions may be due to similarities in the behavior of the therapists, which we did not measure directly; if so, this behavior certainly is based on quitediverging theoretical considerations” (p. 610) “At the postmeasurement, the effects of the psychodynamic therapy seem fewest” (p. 609) “It is striking that in psychodynamic therapy the effects on the intrusion dimension of the Impact of Event Scale clearly lag behind those on the avoidance dimension” (p. 610) “Both other forms of therapy, most notably trauma desensitization, strive to bring about confrontations with images … In this regard the therapy forms substantially differ from one another, and this is mirrored in the results” (p. 610).

*Schnurr et al. (2003)*	
1 “no overall difference between therapy groups on any outcome” (p. 481)	*The full quote reads as follows.* “…intention-to-treat analyses found no overall differences between therapy groups on any outcome. Analyses of data from participants who received an adequate dose of treatment suggested that trauma-focused group therapy reduced avoidance and numbing and, possibly, PTSD symptoms.”

*McDonagh et al. (2005)*	
1 “treatments did not differ significantly at any assessment time point on any measure” (p. 519) 2 “no significant differences” (p. 519)	*Quote 1 is not on p. 519.* *Relevant quote on p. 520:* “Our hypothesis that CBT would be superior to PCT in promoting recovery received support from the finding that CBT was superior to PCT in achieving remission from the PTSD diagnosis at follow-up. …the two active treatments did not differ significantly at any assessment time point on any other measure” (p. 520) *Other relevant quote:* “CBT participants were significantly more likely than PCT participants to no longer meet criteria for a PTSD diagnosis at follow-up assessments” (p. 515)

## Footnotes

1 In addition, two guidelines also recommend stress-inoculation training (Foa, Keane, Friedman & Cohen, 2005; Veterans Health Administration & Department of Defense, 2004) and one guideline states that clinical consensus suggests that psychodynamic therapy is useful in PTSD although this treatment “has not been well studied by means of randomized, controlled trials” (American Psychiatric Association, 2004, p. 52).

2 This review only judged the evidence sufficient for the subset of these treatments that include a significant amount of systematic exposure to trauma memories and reminders (classified as exposure therapies).
